# Efficacy of pioglitazone in correcting ovulatory functions among polycystic ovary syndrome patients

**DOI:** 10.6026/973206300223385

**Published:** 2026-06-30

**Authors:** Ulvina Khalid, Anam Rashid, Vidhi Janak Shah, Mehwish Saleem, Sadia Detho, Rabia Khursheed

**Affiliations:** 1 Department of Obstetrics and Gynaecology, Integrated Medical Care Hospital, Lahore, Pakistan; 2 Department of Obstetrics and Gynaecology, North Manchester General Hospital, Manchester, England; 3 Department of Obstetrics and Gynaecology, Aster Clinic, Dubai; 4 Department of Obstetrics and Gynaecology, Government Mian Meer DHQ level Hospital, Lahore, Pakistan; 5 Department of Obstetrics and Gynaecology, Murshid Hospital, Karachi, Pakistan; 6 6Department of Obstetrics and Gynaecology, Services Hospital, Lahore, Pakistan

**Keywords:** Polycystic ovary syndrome, pioglitazone, menstrual cycle and ovarian function

## Abstract

Polycystic ovary syndrome (PCOS) is a major cause of infertility due to chronic anovulation, and current treatments like metformin are not effective for all patients. Evidence on pioglitazone's role in restoring ovulation is limited, particularly among South Asian women where PCOS prevalence is high. This study, conducted in Lahore from January to June 2025, evaluated pioglitazone in 95 PCOS patients. After six months of treatment, 72.6% of patients achieved restored ovulation with improved hormonal profiles. Pioglitazone thus emerges as an effective alternative to metformin, improving menstrual cycles, ovulation, hyperandrogenism, and reducing the risk of type 2 diabetes.

## Background:

Polycystic ovarian syndrome is one of the most prevalent endocrine conditions in women of reproductive age (PCOS). PCOS is more common in some ethnic groups than others, and it seems to get less common as people get older. According to estimates, the prevalence is 33% among British women between the ages of 20 and 25 [1]. It is 21.6% among Finnish women under the age of 36 [2] and 21% in Indian [3] among women of reproductive age. Compared to white people in the UK (20-25%), South Asian women, particularly Pakistani and Indian women, have a substantially greater frequency of PCOS (52%) [4, 5]. Based on their medical histories, physical characteristics, and research, women with PCOS can be divided into four possible phenotypes [8]. Oligo-/anovulation + polycystic ovaries + hyperandrogenism (PCOS Complete) is the first type. Polycystic ovaries + oligo-/anovulation, polycystic ovaries + hyperandrogenism, and oligo-/anovulation + hyperandrogenism are the other three [9]. Measurements of the number, size, and volume of antral follicles are frequently made using a transvaginal ultrasonogram. According to the Rotterdam diagnostic criteria, the existence of 12 follicles that range in size from 2 to 9 mm and an ovarian volume greater than 10 ml are considered to be typical polycystic ovarian morphology (PCOM) [6, 9]. According to the 2003 Rotterdam criteria, a greater prevalence rate (almost 50%) was discovered in Pakistani females in a study conducted in 2015 by Akram and Roohi [5, 10, 11]. Around 40% of infertile females attend medical health clinics in Pakistan, according to other studies from that country [5, 6, 7, 8, 9, 10, 11, 12, 13]. Regardless of weight, insulin resistance with hyperinsulinemia is present in over 70% of PCOS patients [14]. Pioglitazone is a thiazolidinedione derivative used to treat type 2 diabetes via the PPAR-r (peroxisome proliferator-activated receptor) pathway. Pioglitazone reduces peripheral insulin resistance [14]. Recently, it has been suggested that CC-resistant women with PCOS use insulin-sensitizing medications such as metformin and pioglitazone to induce ovulation. Research has shown that pioglitazone directly decreases the generation of estradiol and testosterone in human ovarian cells. They also improve clinical, hormonal, and metabolic status in PCO patients. Patients with PCOS may benefit from pioglitazone's direct impact on the ovaries and the stimulation of a decrease in peripheral insulin resistance [1]. Therefore, it is of interest to report the efficacy of pioglitazone among PCO patients. Since there is a dearth of available published work on the subject, the findings of this study will be helpful for the use of pioglitazone among PCO patients.

## Methodology:

A total of 95 patients were included. We recorded the BMI, height, weight, age, and menstrual history. Baseline tests were also sent to the laboratory for CBC, LFT, UC, hormonal profile, FSH, LH, prolactin, testosterone, SHBG, Day 21 Progesterone, TSH, and estradiol level on day 2 or 3, calculating the free androgen index. Inducted patients went for pelvic ultrasonography for follicular size on cycle day 8 for antral follicle count and ovarian volume and on day 12 to observe mature graffian follicles of 20 mm size. Patients were given pioglitazone 30 mg in OD dose for 3- 6 months, depending on their ovulatory function, and they were asked to maintain a record of their menstrual cycle. Patients were called for follow-up after 3 months and results were assessed by resumption of the monthly menstrual cycle. Afterwards, all baseline investigations were repeated at 6 months period findings were compared with baseline tests and information were entered in already designed Performa. In the socio-demographic data, descriptive statistics, including mean and standard deviation, were used to present quantitative data. The frequency and percentages were used to convey qualitative factors. A chi-square test was used after stratification, with a p-value of ≤ 0.05 considered significant.

## Results:

In the present study, the age of the study subjects ranged from 19 to 39 years (mean 27.9 ±4.2). Whereas, mean height, weight, BMI, FSH, LH, prolactin,day21 progesterone, fasting insulin level, and free androgen index in our study was 153.5±5.3 cm, 64.9±13.8 kg, 29±4.8 kg/m2, 5.4±1.4 μiu/ml, 10.7±3.1 μiu/ml, 21.5±1.7 ng/ml, 16.2±2.1 μiu/ml,10.9±2.2 μiu/ml and 6.8±2.0 respectively. As shown in Table 1. Out of 95 patients with PCOS, 69 (72.6%) and 26 (27.4%) achieved and did not achieve efficacy on treatment with pioglitazone. As shown in Figure 1 (see PDF). The frequency distribution of sociodemographic factors is detailed in Table 2. Most of the patients (72.6%) were in the 18-28 age group, the majority (83.1%) were married, and nearly half (48.4%) had a BMI greater than 30 kg/m^2^. As shown in Table 2. The association of the efficacy of pioglitazone with maternal age, marital status, and BMI is detailed in Table 3. The association of pioglitazone effectiveness with the patient's FSH, LH, fasting insulin and day 21 progesterone level is detailed in Table 4.

## Discussion:

Several symptomatic treatment options for Polycystic Ovary Syndrome work through different metabolic pathways. In this study, patients with PCOS were used to assess the effectiveness of pioglitazone. By regulating ovulation and the menstrual cycle, pioglitazone improves peripheral insulin resistance, ovulation and hyperandrogenism in PCOS. Regulation of ovulation may also restore the natural feedback effects of luteal steroids, which would help regulate blood LH levels [16]. A total of 95 PCOS patients were included in this study. The mean age in our study was 27.9±4.2 years. Mean LH, FSH, prolactin, day 21 progesterone, fasting insulin level and free androgen index in our study were, 10.7±3.7 μiu/ml, 5.4±1.4 μiu/ml, 21.5±1.7 ng/ml, 16.2±2.1 μiu/ml,10.9±2.2 μiu/ml and 6.8±2.0 respectively. Out of 95 patients with PCOS, 69 (72.6%) and 26 (27.3%) achieved and did not achieve efficacy on treatment with pioglitazone. Compared to our findings, Sahmay *et al.* showed that although there were no statistically significant differences between the subgroups [17]. 30% and 45.5% of the patients demonstrated improvement in menstrual cycle regularity immediately after finishing treatment and 6 months after finishing pioglitazone medication, respectively There was a significant difference between the subgroups in the body mass index (BMI) variation during the course of the study (p = 0.008). The level of free testosterone dropped, but no noticeable change was observed. None of the insulin resistance indicators showed a statistically significant improvement, however baseline free testosterone levels were associated with an increase in the HOMA insulin sensitivity score (p = 0.0009) [18]. Pioglitazone treatment significantly enhanced a number of insulin action measurements when compared to placebo, including glucose disposal rate (P <0.01), 2-hour glucose during a 75-gram oral glucose tolerance test (P < 0.01), the area under the glucose curve during the test (P < 0.01), serum adiponectin (P <0.01), and fasting hyperinsulinemia (P < 0.01). Pioglitazone treatment decreased the increase of leuprolide-stimulated 17-OHP compared to placebo (P < 0.02). Reduced 17-OHP stimulation was linked with increased glucose disposal rate (P < 0.02) [19]. In a different meta-analysis, searches turned up 486 relevant publications including 11 research publications. The overall risk estimates were computed using fixed effects and random effects models. According to the meta-analysis findings, the analysis's pioglitazone treatment group's improvement of the menstrual cycle and ovulation was superior to the metformin group's [OR = 2.31, 95% CI (1.37, 3.91), P < 0.001, I 2 = 41.8%]. The metformin therapy group improved the F-G scores more than the pioglitazone group [SMD = 0.29, 95% CI (0.0, 0.59), P = 0.048, I 2 = 0.0%]. The other data did not significantly differ between the two groups. According to this meta-analysis, pioglitazone improved menstrual cycle and ovulation more than metformin did, whereas metformin improved BMI and F-G scores more than pioglitazone did when treating PCOS patients. It was determined that Pioglitazone might be an effective therapy option for PCOS patients who were unable to tolerate or benefit from metformin [20]. A randomized placebo-controlled experiment was carried out by Aroda *et al.* on a total of 23 PCOS patients [21], who were assessed at baseline and at the conclusion of the trial at the University of California, San Diego. In his study, PCOS patients were randomly assigned to receive an oral placebo or 45 mg of pioglitazone every day for six months. In view of the research linking PCOS and diabetes to CV risks, it is prudent for clinicians to routinely assess women with diabetes for PCOS and to focus appropriate therapy on minimizing associated risks. This study provides novel evidence on the efficacy of pioglitazone in restoring ovulatory function among South Asian women with PCOS, a population with a disproportionately high prevalence and metabolic risk [5]. Previous literature has emphasized metformin as the primary insulin-sensitizing agent; however, our findings demonstrate that pioglitazone significantly improves ovulatory outcomes and menstrual regularity [21]. This contributes to the growing body of evidence supporting pioglitazone as an alternative therapy in cases of metformin intolerance or resistance, and offers region-specific data that may guide clinicians in tailoring treatment strategies [7]. Furthermore, the study underscores the importance of evaluating pharmacological interventions beyond metformin, thereby advancing knowledge in individualized PCOS management.

## Conclusion:

Pioglitazone effectively restores menstrual cycles, improves ovulation, reduces hyperandrogenism and lowers diabetes risk in PCOS patients. It is a suitable alternative when metformin is not tolerated or effective in insulin resistant or obese women. Lifestyle measures such as exercise and weight loss remain essential to reduce cardiovascular and metabolic risks.

## Figures and Tables

**Table 1:** Descriptive Statistics (n=95)

**VARIABLE**	**MEAN ± SD**	**MIN-MAX**
Age	27.9± 4.2	19-39
Height	153.5±5.3	145-165
Weight	64.9±13.8	40-89
BMI	29±4.8	19-35
FSH	5.4±1.4	8-Feb
LH	10.7±3.7	15-Feb
Prolactin	21.5±1.7	18-25
Fasting insulin	10.9±2.2	14-Mar
Day 21 progesterone	16.2±2.1	14-22
Free androgen index	6.8±2.0	9-Feb

**Table 2:** Frequency distribution of sociodemographic factors

**Characteristic**	**N**	**%**
Age (Years)		
18 - 28	69	72.6
29 - 39	26	27.4
BMI		
18-24.9	11	11.6
25-29.9	38	40
>30	46	48.4
MARITAL STATUS		
YES	79	83.2
NO	16	16.8

**Table 3:** Association of efficacy of pioglitazone with Age, BMI and marital status

**Demographics**	**Efficacy of PIOGLITAZONE**		**P-value**
	Yes (n=69)	No (n=26)	
Age (Years)			
18 - 28	51 (74%)	18 (69%)	0.415
29 - 39	18 (26%)	08 (31%)	
BMI			
18-24.9	9(13%)	2(8%)	0.441
25-29.9	25(36%)	13(50%)	
>30	35(51%)	11(42%)	
MARITAL STATUS			
YES	57(83%)	22(85%)	0.542
NO	12(17%)	4(15%)	

**Table 4:** Difference of Efficacy of Pioglitazone with LH, fasting insulin, serum FSH and day 21 progesterone Serum Prolactin and free androgen index

**Characteristics**	**Efficacy of PIOGLITAZONE**		**Mean Diff (P-value)**
	Yes (n=69)	No (n=26)	
Serum LH	12.7 ± 1.84	5.5 ± 2.23	7.2 (< 0.001**)
Fasting Insulin	12.03 ± 0.84	7.96 ± 2.3	4.07 (< 0.001**)
Serum FSH	5.39±1.43	5.77±1.36	0.378 (0.250)
D21 P4	15.04±0.49	19.27±1.58	4.22 (< 0.001**)
Prolactin	21.54±1.64	21.50±1.98	0.036 (0.968)
FAI	8.07±0.35	3.73±0.83	4.34 (< 0.001**)
